# Base deficit and SOFA score are predictive factors of early acute kidney injury in oncologic surgical patients

**DOI:** 10.1186/cc14377

**Published:** 2015-03-16

**Authors:** A Gerent, J Almeida, E Almeida, A Lousada, C Park, J Ribeiro, J Fukushima, A Leme, E Osawa, A Rezende, I Bispo, F Galas, L Hajjar

**Affiliations:** 1 Instituto do Cancer do Estado de São Paulo - ICESP, São Paulo, Brazil

## Introduction

Patients who undergo major oncology surgery are under high risk to develop postoperative acute kidney injury (AKI), mainly due to inflammatory and ischemic insults. This complication results in worse outcomes. The aim of this study is to identify predictive factors of AKI in this population.

## Methods

We performed an observational study in 285 consecutive patients admitted to a surgical ICU after major abdominal oncology surgery. Baseline characteristics, laboratorial, clinical and intraoperative data, such as type of fluids, blood transfusion, bleeding and use of vasopressor, were collected at ICU admission. Early acute kidney injury was defined according to the Acute Kidney Injury Network classification at 48 hours of ICU admission. Logistic regression model was performed using AKI as the outcome.

## Results

There were 76 (26.7%) patients who developed AKI within the first 48 hours after ICU admission. In a univariate analysis, patients with AKI were more likely to be male, had higher Sequential Organ Failure Assessment (SOFA) score, higher baseline serum creatinine and urea levels, higher serum lactate levels and had more metabolic academia at admission. These patients also had a higher 24-hour Simplified Acute Physiology III score and higher length of mechanical ventilation as compared with non-AKI patients. There were no differences between patients regarding intraoperative vasopressors, type and amount of fluids, diuresis and blood transfusion. In a multivariate analysis we identified admission base deficit (BD) (OR = 1.13, 95% CI = 1.02 to 1.24, *P = *0.017) and SOFA score (OR = 1.35, 95% CI = 1.2 to 1.51, *P *< 0.001) as independent predictive factors of early AKI.

## Conclusion

Both SOFA score and BD may be used to predict AKI in surgical oncology patients at ICU admission. These variables allow physicians to recognize early patients who might be under risk, and anticipate measures to avoid further renal impairment.
